# Amyloid-Like Fibril Formation by PolyQ Proteins: A Critical Balance between the PolyQ Length and the Constraints Imposed by the Host Protein

**DOI:** 10.1371/journal.pone.0031253

**Published:** 2012-03-09

**Authors:** Natacha Scarafone, Coralie Pain, Anthony Fratamico, Gilles Gaspard, Nursel Yilmaz, Patrice Filée, Moreno Galleni, André Matagne, Mireille Dumoulin

**Affiliations:** 1 Laboratory of Enzymology and Protein Folding, Centre for Protein Engineering, Institute of Chemistry, University of Liège, Liège, Belgium; 2 Biological Macromolecules, Centre for Protein Engineering, Institute of Chemistry, University of Liège, Liège, Belgium; National Institute for Medical Research, Medical Research Council, London, United Kingdom

## Abstract

Nine neurodegenerative disorders, called polyglutamine (polyQ) diseases, are characterized by the formation of intranuclear amyloid-like aggregates by nine proteins containing a polyQ tract above a threshold length. These insoluble aggregates and/or some of their soluble precursors are thought to play a role in the pathogenesis. The mechanism by which polyQ expansions trigger the aggregation of the relevant proteins remains, however, unclear. In this work, polyQ tracts of different lengths were inserted into a solvent-exposed loop of the β-lactamase BlaP and the effects of these insertions on the properties of BlaP were investigated by a range of biophysical techniques. The insertion of up to 79 glutamines does not modify the structure of BlaP; it does, however, significantly destabilize the enzyme. The extent of destabilization is largely independent of the polyQ length, allowing us to study independently the effects intrinsic to the polyQ length and those related to the structural integrity of BlaP on the aggregating properties of the chimeras. Only chimeras with 55Q and 79Q readily form amyloid-like fibrils; therefore, similarly to the proteins associated with diseases, there is a threshold number of glutamines above which the chimeras aggregate into amyloid-like fibrils. Most importantly, the chimera containing 79Q forms amyloid-like fibrils at the same rate whether BlaP is folded or not, whereas the 55Q chimera aggregates into amyloid-like fibrils only if BlaP is unfolded. The threshold value for amyloid-like fibril formation depends, therefore, on the structural integrity of the β-lactamase moiety and thus on the steric and/or conformational constraints applied to the polyQ tract. These constraints have, however, no significant effect on the propensity of the 79Q tract to trigger fibril formation. These results suggest that the influence of the protein context on the aggregating properties of polyQ disease-associated proteins could be negligible when the latter contain particularly long polyQ tracts.

## Introduction

Polyglutamine (polyQ) diseases are neurodegenerative disorders caused by the expansion of unstable CAG trinucleotide repeats in the translated region of unrelated genes. These CAG repeats encode a polyglutamine stretch in the corresponding proteins [1]. At least nine polyQ-related disorders are known including Huntington's disease and several spinocerebellar ataxias [1]. The nine disease-associated proteins show no sequence or structural similarity apart from the expanded polyQ tract which is located at a different position in each protein. The polyQ tract appears, therefore, to be a critical determinant of polyQ diseases and several lines of evidence suggest that it confers a toxic function to the mutant proteins [2], [3]. Although the nine diseases present distinct pathological and molecular phenotypes, they share a number of common features, suggesting a common physiopathological mechanism [4]. Firstly, there is a threshold in the number of repeats above which polyQ proteins become pathogenic. The value of this threshold varies from one disease to another but generally resides between 35 and 45 glutamines [5]. Secondly, above the threshold value, the longer the repeat, the earlier the onset and more severe the disease; this phenomenon is known as the “anticipation phenomenon” [4]. Thirdly, polyQ expansion mediates the deposition of nuclear inclusion bodies that contain amyloid-like fibrils [6]. Although the mechanism of toxicity associated with pathological expansion of polyglutamine tracts remains unclear, a large body of evidence indicates that it is associated with protein misfolding and aggregation [7], [8]. Thus, the cytotoxicity of proteins containing an expanded polyQ tract has been attributed to: (i) the formation of inclusion bodies [9], [10], (ii) the presence of misfolded protein monomers [11] and (iii) the transient formation of oligomers during the aggregation process [12], [13], [14].

Two major scenarios, which are not necessarily mutually exclusive, have been put forward to explain how expanded polyQ tracts promote the aggregation of proteins [5], [15]. Firstly, it has been suggested that long polyQ repeats (>36Q) have a high intrinsic propensity to form “polar zippers” which consist of β-sheets stabilized by hydrogen bonds between both main-chain and side-chain amides [16]. The formation of such structures is thought to trigger aggregation into amyloid fibrils. Alternatively, expanded polyQ tracts have been suggested to destabilize the proteins and thereby facilitate the formation of partially unfolded species [17], [18], [19]. Such species generally expose at least part of their main chain and hydrophobic residues to solvent and are therefore prone to intermolecular interactions leading to fibril formation. Such a mechanism has been described for other proteins associated with amyloidosis, including transthyretin [20] and human lysozyme [21], [22].

Although the aggregation of polyQ proteins critically depends on the expansion of the polyQ stretch above the pathological threshold, it is however becoming increasingly evident that regions outside the polyQ tract can modulate both the kinetics and the pathway of aggregation [15], [23]. The observation that the aggregation threshold of polyQ peptides is significantly lower (≥15Q) than that observed for the proteins associated with diseases is a clear indication of the effects of the surrounding sequences [16], [24]. Another evidence was provided by Bhattacharyya and co-workers [23], who showed that the addition of ten prolines at the C-terminal end of polyQ peptides decreased both their rate of *in vitro* aggregation and the stability of the resulting aggregates. The influence of the flanking sequences was also observed in the context of different proteins [25], [26], [27].

Various mechanisms have been proposed to explain the influence of the polyQ flanking regions on the aggregation properties of polyQ proteins [5]. Host protein domains could protect against aggregation by: (i) enhancing total protein solubility [27], [28], [29], (ii) sterically hindering polyQ intermolecular interactions and/or (iii) restricting the polyQ conformational changes required for fibril formation [23], [30]. On the other hand, host protein domains adjacent to the polyQ tract can assist aggregation by providing additional aggregation-prone regions. There is indeed experimental evidence that at least ataxin-3 and the first exon of huntingtin (Htt exon 1) form fibrillar aggregates via a complex multidomain mechanism initiated by intermolecular interactions within non-polyQ regions of the protein. Although the polyQ region is not directly involved in this step, it does indirectly modulate the aggregation propensity of the non-polyQ region through a mechanism that is yet to be fully understood. It possibly involves the destabilization of the non-polyQ region and/or changes in its structure or dynamics [19], [31]. At later stages, the expanded polyQ tract is then directly involved in the formation of the core of mature fibrils [17], [19], [32].

Taken together, these results highlight the existence of a complex interplay between the intrinsic properties of the polyQ tract to trigger aggregation and the modulating effects of the host protein. To gain a better insight into the general principles governing this complex phenomenon, it is necessary to investigate in detail which properties of the host protein influence the ability of polyQ stretches to mediate aggregation; this is therefore the subject of intensive research [17], [19], [23], [30], [32]–[37]. The difficulty in handling proteins involved in diseases, essentially due to their large size and insoluble character, has prompted the design and use of model proteins [11], [18], [25], [31], [34], [35], [37]–[40]. The characterization of these proteins unambiguously points to the length and the location of the polyQ tract as important factors [18], [34], [35], [37]. A number of questions are, however, not yet fully addressed; these include the role of the sequence, size, structure, stability, dynamics and topology of the host protein. There is therefore a clear need for the generation of new model polyQ proteins of various sizes, topologies and structures and the extensive characterization of their structural, thermodynamic, dynamic and aggregating properties.

In proteins associated with polyQ diseases, the polyQ tract is separated from the C- or N-terminal ends by at least 50 residues, except for the proteins associated with Hungtinton's disease (HD, 17 residues) and spinocerebellar ataxia type 6 (SCA6, 30 residues) [34]. Many model proteins characterized so far, however, display a polyQ tract fused to their N- or C-terminus [35], [37], [39], [41], probably because polypeptide chains with an inserted polyQ sequence are difficult to express [35], [37]. It is therefore essential to investigate more model proteins containing an inserted polyQ stretch since the presence of flanking sequences at both extremities of the polyQ tract is likely to impose constraints that are more physiologically relevant than when the polyQ tract has a free extremity. With this aim in mind, we inserted polyQ sequences of various lengths into a solvent-exposed loop of a well-characterized globular protein, and we investigated the effects of these insertions on its structure, stability and aggregating properties.

**Figure 1:**
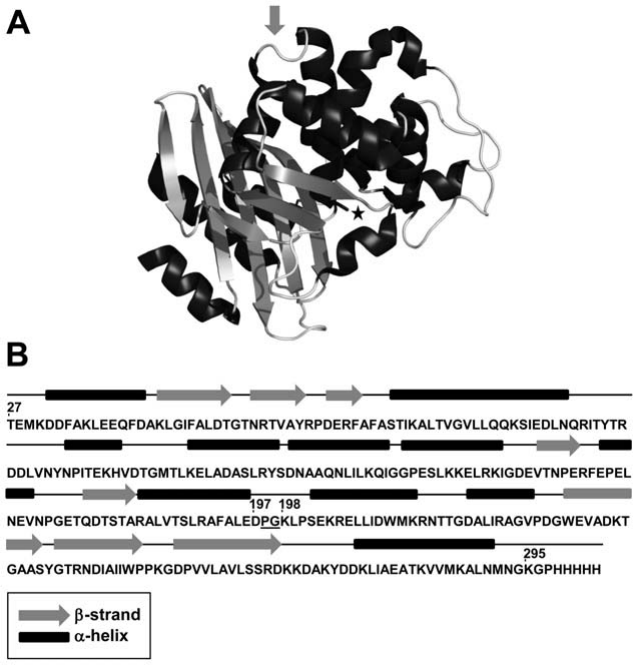
X-ray structure and topology of the host protein BlaP. (**A**) The structure of BlaP was produced using PyMOL (DeLano Scientific LLC, South San Francisco, CA, USA) and the PDB ID is 4BLM [42]. The active site serine (Ser70) is indicated by a star and the insertion site is shown by an arrow. (**B**) Topology of BlaP. The position 197–198 of the insertion site refers to the numbering scheme of class A β-lactamases [65]. The underlined PG dipeptide between residues 197 and 198 corresponds to the SmaI restriction site inserted into the gene of BlaP for the cloning of CAG repeats.

The β-lactamase from *Bacillus licheniformis* 749/C (BlaP) was used as the protein scaffold. This 264-residue enzyme is organized into two structural domains, with the active site located at the interface of the two domains (Figure 1A) [42]. We chose this scaffold for several reasons: (i) detailed information concerning its thermodynamic and catalytic properties are available [43], [44], providing a strong basis to investigate the effects of polyQ insertions, (ii) the solvent-exposed loop located between helices 8 and 9 (the position 197–198, Figure 1A) has been clearly shown to tolerate amino acid insertion [44], [45], and (iii) chimeras with inserts of various lengths and structures can be readily produced in *Escherichia coli* ([44], [45] and unpublished results).

We have created and characterized a series of chimeras with 23, 30, 55 and 79 glutamines inserted at position 197–198 (Figure 1A and B) using a range of biophysical techniques including fluorescence, circular dichroism (CD), transmission electron microscopy (TEM) and X-ray diffraction. We have found that the insertion of a polyQ tract consisting of up to 79 residues does not modify the structure of the enzyme, although it significantly reduces its conformational stability. The results of this study also show that the aggregating properties of the chimeras are similar to those of proteins associated with polyQ diseases. Thus, we observed a threshold number of inserted glutamines above which the chimeric β-lactamase aggregates into amyloid-like fibrils and that the kinetics of aggregation are faster with longer glutamine repeats. Most importantly, this work clearly demonstrates that the threshold number of glutamines above which the chimeric proteins aggregate into amyloid-like fibrils critically depends on the structural integrity of the β-lactamase moiety and thus on the constraints applied to the polyQ tract. It also suggests that the modulating effects of the protein context on the aggregating properties of proteins associated with polyQ diseases could be negligible when the latter contain particularly long polyQ tracts. Finally, our results indicate that BlaP is a promising scaffold for further investigation of the delicate balance between the propensity of polyQ tracts to trigger aggregation and the modulating effects of the host proteins.

## Results and Discussion

### Production of the chimeras

**Figure 2:**
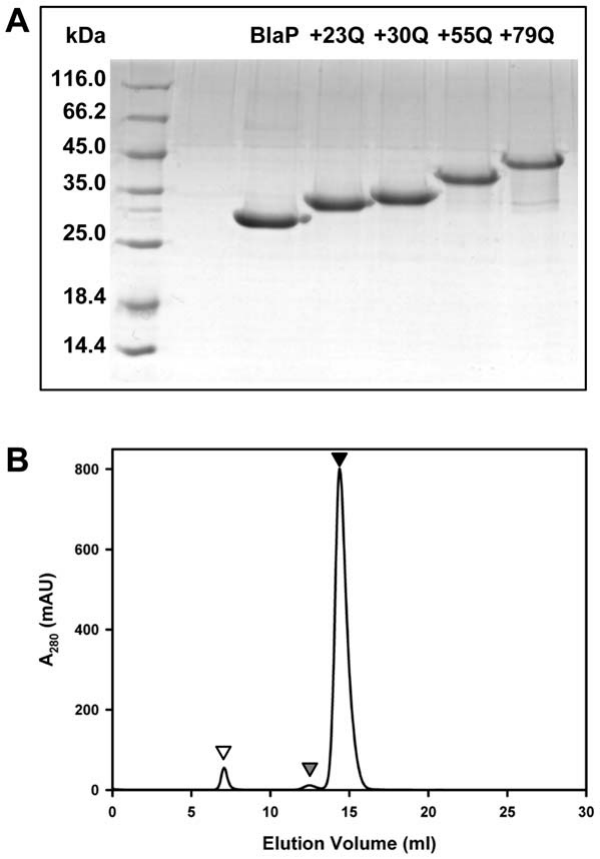
Purification of BlaP and the chimeras. (**A**) Purified BlaP and polyQ-containing chimeras separated on a 15% (w/v) SDS-polyacrylamide gel and stained with coomassie blue. First lane on the left shows protein markers with molecular masses as indicated, whereas the other lanes show BlaP and the various chimeric proteins as indicated. Expected molecular masses are 30 368, 33 315, 34 212, 37 416 and 40 491 Da for BlaP and chimeras with 23, 30, 55 and 79 glutamines, respectively. (**B**) SEC analysis of BlaP(Gln)_79_ at 120 µM in PBS, pH 7.5; mAU, milli-absorbance units. Monomeric, dimeric and high molecular weight species are indicated by black, grey and white arrows, respectively. The high molecular weight oligomeric species are eluted in the void volume of the column.

Four expression vectors encoding for chimeric β-lactamases containing 23, 30, 55 and 79 glutamines [BlaP(Gln)_23_, BlaP(Gln)_30_, BlaP(Gln)_55_ and BlaP(Gln)_79_, respectively] were constructed as described in the Materials and Methods, by inserting CAG repeats into a SmaI restriction site incorporated into the BlaP gene between codons for residues 197 and 198. The introduction of this SmaI restriction site adds a dipeptide proline-glycine to the sequence of BlaP; the polyglutamine tract was inserted between these two amino acids (Figure 1B). The proteins were produced in *E. coli* and purified to homogeneity in one step using a Ni-PDC affinity column. This procedure led to about the same amount (i.e. 10–20 mg per liter of culture) of the different proteins, irrespective of the presence or absence of a polyQ tract. The purity of all samples was >95% as assessed by SDS-PAGE (Figure 2A). Although BlaP, BlaP(Gln)_23_ and BlaP(Gln)_30_ migrated at positions expected, according to the molecular mass markers, chimeras with either 55 or 79 glutamines showed lower mobility and thus, greater apparent molecular masses than expected. Such anomalous behaviour, which was also observed for chimeras composed of myoglobin and polyQ tracts [40], suggests that long polyQ sequences interfere with SDS binding. The integrity of the proteins was checked by mass spectrometry (ESI-QTOF-MS). This analysis revealed the presence of three distinct peaks in the spectrum of each protein. Although in all cases, the main peak (estimated at >70% of the population) corresponds to the theoretical mass deduced from the complete amino acid sequence, the two other peaks correspond to higher molecular mass species, i.e. +182 Da (10–20% of the population) and +200 Da (<10% of the population). N-terminal sequencing indicated that the main peak corresponds to the enzyme with a well-processed N- terminal sequence (T-E-M-K) (Figure 1B), whereas the peak at +200 Da corresponds to the full-length protein plus the last two residues of the signal peptide (Q-A-T-E-M-K). The same N-terminal misprocessing was also observed for another BlaP chimera [44]. The nature of the +182 Da adduct is not clear. Since the proportion of each molecular species is similar between various protein preparations, it is assumed that they do not interfere with the main conclusions of this work.

Size-exclusion chromatography (SEC) analysis of protein preparations at high concentrations (50 µM and 120 µM) revealed that all proteins, particularly BlaP, form dimers although in small amount (at 120 µM, 5.6% for BlaP and <1.5% for the four chimeras, Table 1). Minute quantities of high molecular weight species were also observed in BlaP(Gln)_79_ solution (3.5% at 120 µM and 2.5% at 50 µM) (Table 1 and Figure 2B). Based on their volume of elution and assuming that these oligomeric species are spherical, their apparent molecular mass can be estimated to be higher than 600 kDa, and thus formed by 15 or more monomers. Decreasing the concentration of BlaP(Gln)_79_ samples from 50 µM to 10 µM reduces the percentage of these oligomeric species to less than 2% (Table 1). Following the observation that in all cases more than 95% of the protein population is monomeric at concentrations as high as 50 µM, we assumed that the presence of dimers and oligomers did not interfere with the determination of the catalytic and thermodynamic parameters which were carried out with protein concentrations ≤4.6 µM.

**Table 1:** Percentages of the different species observed by SEC.

	[protein] (µM)	M (%)	D (%)	O (%)
**BlaP**	**50**	95.1	4.9	0
	**120**	94.4	5.6	0
**BlaP(Gln)_23_**	**50**	99.4	0.6	0
	**120**	99.3	0.7	0
**BlaP(Gln)_30_**	**50**	99.6	0.4	0
	**120**	99.5	0.5	0
**BlaP(Gln)_55_**	**50**	98.9	1.1	0
	**120**	98.6	1.4	0
**BlaP(Gln)_79_**	**10**	97.3	0.9	1.8
	**50**	96.4	1.1	2.5
	**120**	95.3	1.2	3.5

This analysis was carried out using a Superdex 200 GL 10/300 column using PBS buffer, pH 7.5. **M**, **D**, **O** stand for monomeric, dimeric, and high molecular weight oligomeric species, respectively.

### The polyQ tract adopts a disordered structure and does not perturb the overall structure of BlaP

**Figure 3:**
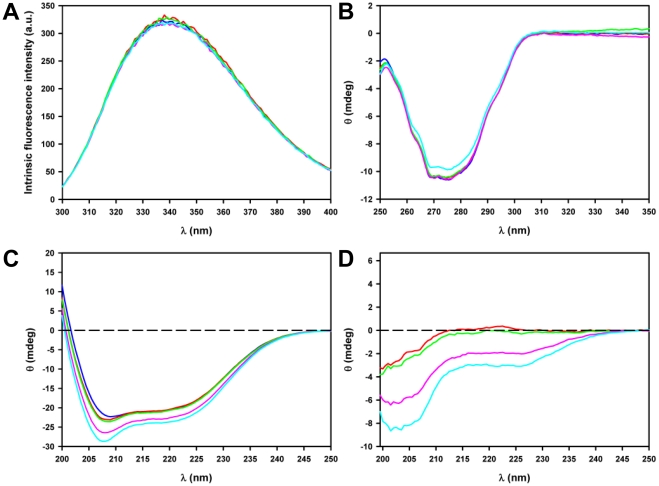
PolyQ insertions have no effect on the structure of BlaP. (**A**) Intrinsic fluorescence, (**B**) near-UV CD and (**C**) far-UV CD spectra of BlaP (blue) and the chimeras with 23 (red), 30 (green), 55 (pink) and 79 (cyan) glutamines. a.u., arbitrary units. (**D**) Difference spectra obtained by subtraction of the far-UV CD spectrum of BlaP from that of each chimeric protein. Spectra were recorded at 25°C in PBS, pH 7.5, using protein concentrations of 4.6 µM (fluorescence and far-UV CD) and 20 µM (near-UV CD).

The effects of the insertion of polyQ tracts of 23, 30, 55 and 79 residues on the structure of BlaP were investigated by intrinsic fluorescence and CD measurements. Fluorescence and near-UV CD data (Figure 3A and B) suggest that none of the polyQ insertions into the loop between the α-helices 8 and 9 of BlaP significantly affects the tertiary structure of the protein. This is further supported by the observation that insertion of polyQ sequences of various lengths does not significantly modify the catalytic properties of the β-lactamase (Table 2). In contrast, the far-UV CD spectra of the chimeras show marked differences with that of the wild-type protein (Figure 3C). Subtraction of the BlaP spectrum from the spectra of the chimeras gives an indication of the structure adopted by the polyQ insert. Interestingly, the difference spectra display a negative peak with a minimum at *ca.* 202–203 nm, the amplitude of which increases with the number of glutamines (Figure 3D). This result suggests that the polyQ tract: (i) does not significantly modify the secondary structure of BlaP and (ii) adopts a disordered structure at the surface of the protein irrespective of the number of glutamines.

**Table 2:** Kinetic parameter values for the wild-type and chimeric β-lactamases.

	k_cat_ (s^−1^)	K_m_ (µM)	10^−6^×k_cat_/K_m_ (M^−1^·s^−1^)
**BlaP**	90±10	25±3	3.6±0.4
**BlaP(Gln)_23_**	120±10	32±4	3.7±0.3
**BlaP(Gln)_30_**	87±7	27±3	3.1±0.2
**BlaP(Gln)_55_**	107±10	31±3	3.5±0.5
**BlaP(Gln)_79_**	77±2	27±2	2.9±0.1

Cephalothin was used as the substrate. Errors are given as standard deviations.

A similar absence of structural modification of the non-polyQ regions was observed when a polyQ tract is fused to the B domain of the protein A from *Staphylococcus aureus* (SpA) or apomyoglobin [35], [37]; however, radically different results were reported for inserted polyQ tracts. The most characterized chimeric proteins with inserted polyQ sequences were created from three all-α proteins: myoglobin [18], SpA [35] and apomyoglobin [37]. In these model proteins, the polyQ tract was inserted into a loop between α-helices. The insertion of a polyQ tract into either myoglobin [18] or SpA [35] induces changes in the tertiary structure of the host proteins, with no significant effect on their secondary structure. The insertion of at least 16Q in apomyoglobin leads, however, to significant changes in secondary structure, besides a polyQ length-independent loss of tertiary structure [37]. This partial loss of structure was also observed when serine-glycine repeats were inserted at the same location, suggesting that it was not specific to polyQ insertion. As a likely consequence of these structural changes, the production yield for at least two of these model proteins was significantly lower than that of their respective wild-type counterparts, and this was shown to be directly related to the length of the polyQ tract inserted [35], [37]. To date, the model protein with the longest inserted polyQ sequence reported in the literature consists of a chimeric myoglobin containing 50 glutamines only [18]. In contrast, the production yield of our BlaP chimeras remains unchanged (i.e. 10–20 mg per liter of culture), even when a 79-glutamine sequence is inserted at position 197–198. This makes BlaP an ideal scaffold to investigate the effects of long inserted polyQ sequences.

Our CD data suggest that the polyQ tracts of 23-79Q, inserted into the solvent-exposed loop of BlaP, adopt a disordered structure. This observation is in very good agreement with the results obtained with other model proteins containing variable-length polyQ tracts, e.g. (i) polyQ sequences fused either to SpA or GST [35], [39], [41] and (ii) the exon 1 of huntingtin fused to thioredoxin [38]. In contrast, data obtained with myoglobin and apomyoglobin indicated that inserted polyQ sequences of sufficient length (*ca.* 16-28Q) could form β-structures [18], [37]. These findings suggest that the conformation adopted by a polyQ stretch is strongly context-dependent. The observation that a 79-residue long polyQ sequence is disordered when inserted into BlaP, suggests that it does not adopt any stable, aggregation-prone, β-sheet structure in solution prior to aggregation into amyloid-like fibrils. Because of the low sensitivity of the structural techniques used in this work, we cannot exclude, however, the possibility that the polyQ tract might rarely and/or transiently access more organized structures.

### The polyQ tract destabilizes BlaP and the extent of destabilization is largely independent of the polyQ length

**Figure 4:**
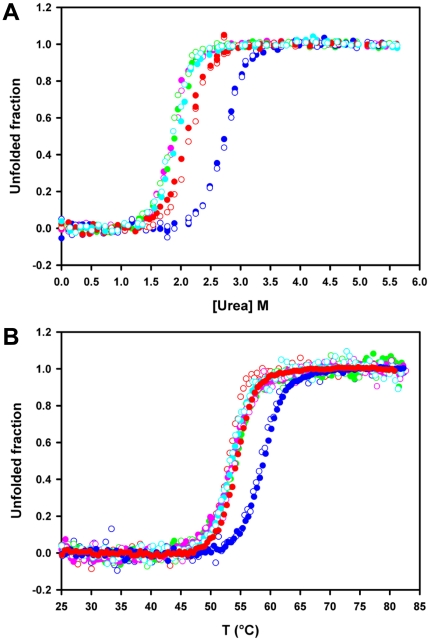
PolyQ insertions destabilize BlaP and the extent of destabilization is largely independent of the polyQ length. (**A**) Normalized urea-induced unfolding transitions at pH 7.5 and 25°C and (**B**) normalized heat-induced unfolding transitions at pH 7.5, monitored by the changes in fluorescence intensity at 323 nm (filled circles) and in ellipticty at 222 nm (open circles), using a protein concentration of 4.6 µM. BlaP (blue), BlaP(Gln)_23_ (red), BlaP(Gln)_30_ (green), BlaP(Gln)_55_ (pink) and BlaP(Gln)_79_ (cyan). Non-normalized data were analysed on the basis of a two-state model and the values of the thermodynamic parameters obtained are reported in Table 3.

The effects of polyQ insertions of different lengths (23, 30, 55 and 79Q) on the stability of BlaP were determined by urea-induced unfolding experiments. For each protein, unfolding was found to be fully thermodynamically reversible (data not shown) and single transitions were observed (Figure 4A). The data obtained by intrinsic fluorescence and far-UV CD coincide, indicating that the five proteins unfold according to a simple two-state mechanism, with no intermediate species significantly populated between the native and unfolded states. Based on this model, the characteristic thermodynamic parameters were determined and are given in Table 3. These data show that all four chimeras are destabilized with respect to the wild-type protein. Remarkably, the extent of destabilization is similar, i.e. 7.6–8.8 kJ·mol^−1^, for all four chimeric proteins and thus is largely independent of the length of the polyQ stretch. Thermal unfolding of BlaP and its chimeras (Figure 4B) was also monitored by both intrinsic fluorescence and far-UV CD measurements. Although this process was not reversible (data not shown), cooperative transitions were observed and apparent temperatures of mid-transition (T_m_^app^) were determined (Table 3). In good agreement with urea-unfolding data, all chimeras are less stable than the wild-type protein (ΔT_m_^app^ = 4.5–5.3°C) and the extent of destabilization is independent of the number of glutamines.

**Table 3:** Thermodynamic parameter values of unfolding of wild-type and chimeric BlaP.

	ΔG°_NU_(H_2_O) (kJ·mol^−1^)	−*m*_NU_ (kJ·mol^−1^·M^−1^)	C_m_ (M)	T_m_ *^app^* (°C)
**BlaP**	37.0±1.8	13.5±0.6	2.74±0.23	58.5±0.4
**BlaP(Gln)_23_**	29.3±2.4	13.7±0.9	2.13±0.19	53.9±0.7
**BlaP(Gln)_30_**	29.4±1.2	15.9±0.7	1.85±0.15	53.7±0.3
**BlaP(Gln)_55_**	29.0±0.6	15.6±0.6	1.86±0.15	53.5±0.6
**BlaP(Gln)_79_**	28.2±1.3	14.9±0.6	1.89±0.17	53.2±0.3

The values were deduced from the analysis of unfolding transitions monitored by intrinsic fluorescence and far-UV CD measurements. The urea unfolding experiments were carried out at 25°C. **ΔG°_NU_(H_2_O)**, ***m*** **_NU_**, **C_m_** and **T_m_** ***^app^*** values result from the mean of the values obtained by far-UV CD and intrinsic fluorescence; note that for thermal unfolding, three measurements were carried out both by fluorescence and CD. Thermal unfolding was not fully reversible and only the apparent mid-unfolding temperature (T_m_ *^app^*) was determined.

The fact that the extent of BlaP destabilization is independent of the number of glutamines contrasts with observations on other model systems reporting: (i) no destabilization at all, e.g. the protein moeity SpA when fused to a polyQ tract up to 52 residues in length [35], (ii) selective destabilization, e.g. the cellular retinoic acid binding protein I (CRABP I) moiety when fused to Htt exon 1 containing polyQ stretches longer than a threshold size [31], (iii) cumulative destabilization, e.g. chymotrypsin inhibitor 2 (CI2) [46], myoglobin [18] and SpA [35] containing inserted polyQ tracts, where the extent of destabilization increases with increasing polyQ length, independently of a threshold. Finally, a polyQ-length independent destabilization was also described for apomyoglobin containing an inserted polyQ tract [37] but this effect does not seem specific to the glutamines, since a similar destabilization was observed when serine-glycine repeats were inserted at the same location. In the case of BlaP, insertion of the 73-residue long chitin-binding domain of human macrophage chitotriosidase [44] destabilizes the host protein significantly less than the polyQ tracts (i.e. 3.2 kJ·mol^−1^ *vs* 7.6–8.8 kJ·mol^−1^). This suggests that the extent of destabilization of BlaP depends more on the nature of the polypeptide inserted than its length, and that the insertion of the disordered polyQ stretch is more destabilizing than that of the folded chitin-binding domain of human macrophage chitotriosidase. Taken together, these data show that the effects of polyQ insertion on the stability of the host protein vary greatly depending on the properties of the latter.

The observation that the chimeric β-lactamases with 30, 55 and 79 glutamines are destabilized to the same extent relative to wild-type BlaP (Table 3) gave us the unique opportunity to investigate independently the influence of (i) the length of the polyQ sequence and (ii) the conformational state of the β-lactamase moiety on the aggregating properties of the chimeras. Accordingly, the aggregating properties of BlaP and the different chimeras were investigated under both native and denaturing conditions.

### The threshold length of the polyQ tract above which chimeras aggregate into amyloid-like fibrils depends on the structural integrity of BlaP

#### Aggregation under denaturing conditions

**Figure 5:**
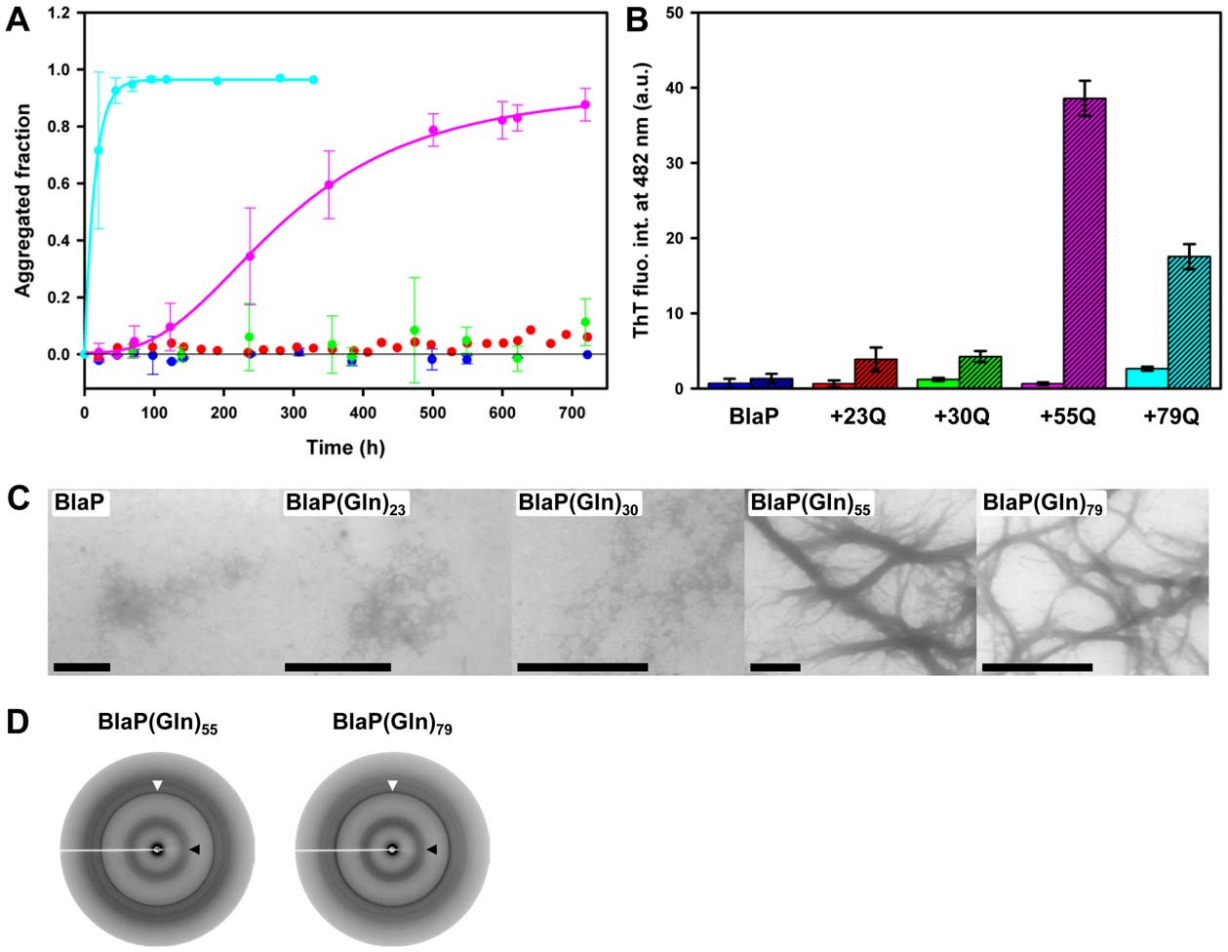
Only BlaP(Gln)_55_ and BlaP(Gln)_79_ form amyloid-like fibrils when incubated in the presence of 1.85 M urea. (**A**) Aggregation kinetics of 110 µM BlaP (blue), BlaP(Gln)_23_ (red), BlaP(Gln)_30_ (green), BlaP(Gln)_55_ (pink) and BlaP(Gln)_79_ (cyan) at 25°C in the presence of 1.85 M urea in PBS, pH 7.5, followed by measuring the concentration of protein remaining soluble. Time-points shown with an error bar are the average of three independent time-courses for BlaP(Gln)_30_, BlaP(Gln)_55_ and BlaP(Gln)_79_ and two independent time-courses for BlaP. Error bars show the standard deviations. Only one time-course was carried out with BlaP(Gln)_23_. (**B**) ThT fluorescence intensities at 482 nm in the presence of BlaP and chimeras samples at T_0_ (solid bars) and T_f_ (dashed bars). T_0_ and T_f_ correspond to the initial and final points of one time-course for each protein. Data are the average of three measurements and error bars represent the standard deviations. a.u., arbitrary units. (**C**) TEM images of the protein samples at T_f_. The scale bar is 1 µm. (**D**) X-ray fibre diffraction patterns from BlaP(Gln)_55_ and BlaP(Gln)_79_ fibrils. White and black arrows indicate meridional and equatorial reflections at 4.7 Å and *ca.* 9.5 Å, respectively.

The aggregating properties of the proteins were first investigated in the presence of 1.85 M urea (in PBS, pH 7.5), at 25°C. Data in Figure 4A and Table 3 indicate that under these conditions, all BlaP molecules are native, whereas *ca.* 18% and 50% of the molecules of, respectively, BlaP(Gln)_23_ and the three other chimeric enzymes, are unfolded. The kinetics of aggregation were monitored by measuring the amount of protein that remained soluble after different incubation times. BlaP, BlaP(Gln)_23_ and BlaP(Gln)_30_ display little, if any, tendency to aggregate, even after *ca.* 720 hours of incubation, whereas both BlaP(Gln)_55_ and BlaP(Gln)_79_ readily aggregate (Figure 5A). Aggregation of BlaP(Gln)_55_ is characterized by a lag phase (*ca.* 50 hours) while very fast aggregation with no discernable lag phase is observed with BlaP(Gln)_79_. The lag phase is consistent with the nucleation-polymerization mechanism that has been proposed for amyloid fibril formation [47] and corresponds to the nucleation phase. After *ca.* 720 and *ca.* 330 hours of incubation, fibrillar aggregates (Figure 5C) are clearly visible by TEM for BlaP(Gln)_55_ and BlaP(Gln)_79_, respectively. In contrast, even after 720 hours of incubation, only minute amounts of amorphous aggregates, but no fibrils, were observed with BlaP, BlaP(Gln)_23_ and BlaP(Gln)_30_ (Figure 5C). The aggregates formed by BlaP(Gln)_55_ and BlaP(Gln)_79_ after, respectively, 720 and 330 hours of incubation significantly bind ThT (Figure 5B), suggesting that the fibrils observed are amyloid-like. The degree of ThT binding observed for BlaP(Gln)_79_ is, however, significantly lower than that observed for BlaP(Gln)_55_, a surprising observation considering that BlaP(Gln)_79_ aggregates to a higher extent than its 55-glutamine homolog (Figure 5A). The significantly lower ThT binding to BlaP(Gln)_79_ fibrils may be due to an alternative overall packing of the aggregates. This is consistent with the observation that aggregates formed by BlaP(Gln)_79_ were clearly larger and more difficult to resuspend in the ThT solution. For BlaP(Gln)_79_, the ThT fluorescence measurements were performed with samples incubated for *ca.* 330 hours and since the protein is fully aggregated after 100 hours, the aggregates could mature (e.g. laterally associate) during the last 200 hours of incubation. In the case of BlaP(Gln)_55_, the aggregation is much slower and there is therefore less time for fibril maturation until the final point of the time-course (i.e. when samples were taken for ThT fluorescence measurements at *ca.* 720 hours) (Figure 5A). Aggregates formed by BlaP(Gln)_55_ and BlaP(Gln)_79_ were analyzed by X-ray fibre diffraction. Both diffraction patterns obtained show two reflections: a sharp meridional one at 4.7 Å and a broad and more diffuse equatorial one at *ca.* 9.5 Å (Figure 5D). Despite difficulties encountered in aligning the fibrils, these reflections are consistent with a cross-β structure characteristic of amyloid fibrils and reflect the distance between the β-strands in each sheet of the amyloid protofilament (4.7 Å) and the spacing between the β-sheets (*ca.* 9.5 Å) [48].

These results show that, although the three chimeras BlaP(Gln)_30_, BlaP(Gln)_55_ and BlaP(Gln)_79_ are equally destabilized under the conditions used to monitor aggregation (i.e. 50% of the molecules of each protein are unfolded at 25°C in the presence of 1.85 M urea), only those with 55 and 79 glutamines significantly aggregate into amyloid-like fibrils within the time scale of the experiment. Hence, independently of the extent of destabilization, there seems to be a minimal length (i.e. a threshold) of the polyQ tract beyond which chimeric β-lactamases readily aggregate. This value is comprised between 30 and 55 residues, which is reminiscent of the threshold number of glutamines above which other model proteins fused to a polyQ tract aggregate both *in vivo* and *in vitro* [31], [49] and, more importantly, of the pathological threshold observed in polyQ diseases [5].

**Figure 6:**
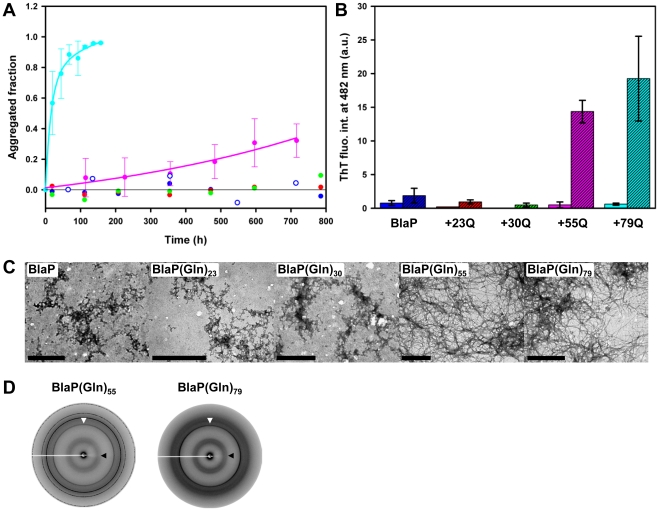
The unfolding of the β-lactamase moiety is not the critical factor that triggers the aggregation process. (**A**) Aggregation kinetics of 110 µM BlaP (blue), BlaP(Gln)_23_ (red), BlaP(Gln)_30_ (green), BlaP(Gln)_55_ (pink) and BlaP(Gln)_79_ (cyan) at 25°C in the presence of 3.5 M urea in PBS, pH 7.5, followed by measuring the concentration of protein remaining soluble. Time-points shown with an error bar are the average of three independent time-courses for BlaP(Gln)_55_ and BlaP(Gln)_79_. Error bars show the standard deviations. For BlaP, two independent experiments were carried out (filled and open blue circles); however, since the times at which samples were taken differ from one time-course to the other, the data could not be averaged. For BlaP(Gln)_23_ and BlaP(Gln)_30_, only one time-course was carried out. (**B**) ThT fluorescence intensities at 482 nm in the presence of BlaP and chimeras samples at T_0_ (solid bars) and T_f_ (dashed bars). T_0_ and T_f_ correspond to the initial and final points of one time-course for each protein. Data are the average of three measurements and error bars represent the standard deviations. a.u., arbitrary units. (**C**) TEM images of the protein samples at T_f_. The scale bar is 1 µm. (**D**) X-ray fibre diffraction patterns from BlaP(Gln)_55_ and BlaP(Gln)_79_ fibrils. White and black arrows indicate meridional and equatorial reflections at 4.7 Å and *ca.* 9.5 Å, respectively.

BlaP and all the chimeras (with 23 to 79Q) were also incubated at 25°C in the presence of 3.5 M urea, a concentration at which all proteins are unfolded (Figure 4A). Under these conditions, again, only BlaP(Gln)_55_ and BlaP(Gln)_79_ noticeably aggregate into fibrils that possess characteristics of amyloid such as ThT binding and cross-β structure (Figure 6A–D). Although BlaP, BlaP(Gln)_23_ and BlaP(Gln)_30_ remain soluble, small amounts of amorphous aggregates are observed by TEM for these proteins; these aggregates do not bind ThT (Figure 6A–C).

Taken together, the results of these two experiments give a clear indication that the unfolding of the β-lactamase moiety is not the driving force underlying the fibrillar aggregation. Rather, it is purely the expansion of the polyQ tract above a threshold length that promotes the formation of amyloid-like fibrils. Moreover, in the presence of 1.85 M or 3.5 M urea, the chimera with 79 glutamines aggregates faster than that containing 55 glutamines. Such a polyQ length-dependent rate of aggregation has been observed in all *in vitro* studies of polyQ peptides [23], [50] and different protein systems [31], [35], and can be related to the so called “anticipation phenomenon” characteristic of polyQ diseases [4].

#### Aggregation under native conditions

**Figure 7:**
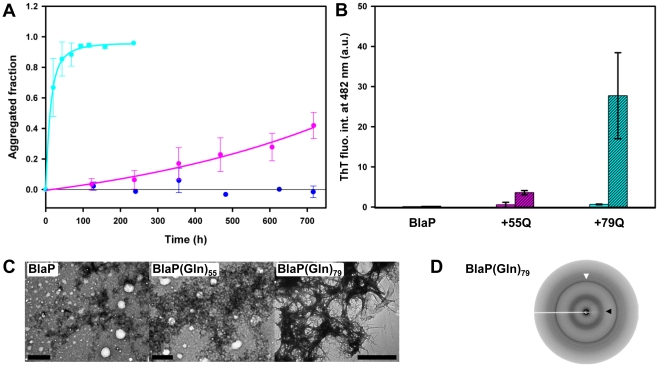
Only BlaP(Gln)_79_ form amyloid-like fibrils when incubated under native conditions. (**A**) Aggregation kinetics of 110 µM BlaP (blue), BlaP(Gln)_55_ (pink) and BlaP(Gln)_79_ (cyan) at 37°C in PBS, pH 7.5, followed by measuring the concentration of protein remaining soluble. Time-points shown with an error bar are the average of three independent time-courses for BlaP(Gln)_55_ and BlaP(Gln)_79_ and two independent time-courses for BlaP. Error bars show the standard deviations. (**B**) ThT fluorescence intensities at 482 nm in the presence of BlaP and chimeras samples at T_0_ (solid bars) and T_f_ (dashed bars). T_0_ and T_f_ correspond to the initial and final points of one time-course for each protein. Data are the average of three measurements and error bars represent the standard deviations. ThT fluorescence intensities in the presence of BlaP samples are weak and for more visibility, error bars (which are equal or less than to 0.3) are not shown. a.u., arbitrary units. (**C**) TEM images of the protein samples at T_f_. The scale bar is 1 µm. (**D**) X-ray fibre diffraction pattern from BlaP(Gln)_79_ fibrils at T_f_. White and black arrows indicate meridional and equatorial reflections at 4.7 Å and *ca.* 9.5 Å, respectively.

**Figure 8:**
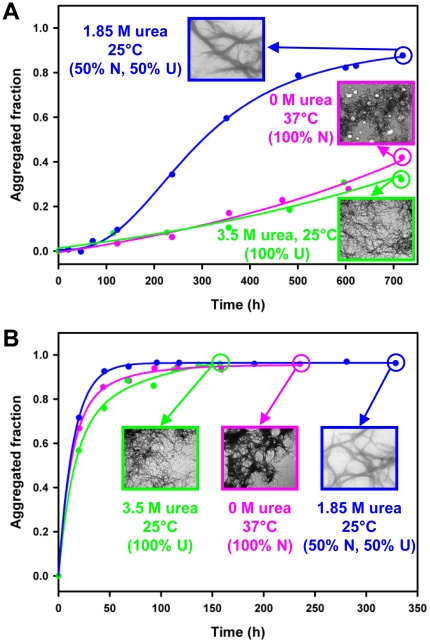
The ability of BlaP(Gln)_79_ to form amyloid-like fibrils does not depend on the structural integrity of BlaP. Comparison between the aggregation kinetics and the morphology of aggregates obtained with BlaP(Gln)_55_ (**A**) and BlaP(Gln)_79_ (**B**) under the following conditions of incubation: (i) PBS, pH 7.5 and 0 M urea at 37°C (pink), (ii) PBS, pH 7.5 and 1.85 M urea at 25°C (blue) and (iii) PBS, pH 7.5 and 3.5 M urea at 25°C (green). N is the native state and U is the unfolded state. The data correspond to those shown in Figures 5, 6, 7 without error bars.

Finally, the kinetics of aggregation of BlaP, BlaP(Gln)_55_ and BlaP(Gln)_79_ were monitored under conditions favouring the native state (PBS, pH 7.5, 37°C) (Figure 4B). As observed under denaturing conditions (Figures 5 and 6), only chimeras with 55 and 79 glutamines aggregate, whereas BlaP remains soluble throughout the duration of the experiment (Figure 7A). The kinetics of aggregation of BlaP(Gln)_79_ was similar to that obtained in the presence of urea (Figures 5A and 6A) and the aggregates formed significantly bind ThT (Figure 7B), display a fibrillar morphology (Figure 7C), and exhibit a X-ray fibre diffraction pattern consistent with a cross-β structure (Figure 7D). These observations are all indicative of amyloid-like fibril formation by BlaP(Gln)_79_ under native conditions. In contrast, BlaP(Gln)_55_ aggregates to a lesser extent than in the presence of 1.85 M urea, and the resulting species bind ThT only weakly (Figure 7B) and appear amorphous when viewed by TEM (Figure 7C). These aggregates did not mature into amyloid-like fibrils upon further incubation of up to 1500 hours (data not shown). Similarly, only small, disperse and amorphous aggregates that do not bind ThT are visible by TEM in BlaP samples at the end of the experiment (Figure 7B and C). These results show that under native conditions, only the chimeric β-lactamase with 79 glutamines forms amyloid-like fibrils. Our data also show that under native conditions, the polyQ threshold length for aggregation into fibrillar aggregates is higher than that observed under denaturing conditions, suggesting that its value depends on the structural integrity of the β-lactamase moiety. Indeed, BlaP(Gln)_55_ is only able to form amyloid-like aggregates when BlaP is unfolded (Figure 8A) whereas, remarkably, BlaP(Gln)_79_ aggregates into amyloid-like fibrils at the same rate irrespective of the incubation conditions and therefore, of the conformational state (i.e. native or unfolded) of BlaP (Figure 8B). This observation shows that the presence of folded BlaP suppresses the intrinsic propensity of the 55-glutamine tract to trigger the self-association of the chimera into amyloid-like fibrils. In other words, in the presence of 55Q, the protective role of BlaP in polyQ-driven aggregation into amyloid-like fibrils depends on the integrity of the BlaP structure. In contrast, the conformational state of BlaP has no discernable effect on the ability of the 79-glutamine tract to promote the formation of amyloid-like fibrils.

The dependence of BlaP(Gln)_55_ aggregation on the structural integrity of BlaP can result from both steric and conformational constraints. The folded β-lactamase moiety may limit the accessibility of the 55-glutamine tract via steric hindrances and thus abrogate its propensity to form the highly ordered intermolecular β-sheets characteristic of amyloid fibrils. The 55-glutamine tract in the presence of folded BlaP leads, nevertheless, to less organized intermolecular interactions and thus to the formation of amorphous aggregates. The unfolding of BlaP can however render the 55Q tract more accessible and thus permits the formation of amyloid-like fibrils. In contrast, when expanded to 79 glutamines, the polyQ tract is similarly accessible to interact with other monomers to form amyloid-like fibrils whether BlaP is denatured or not. The need to unfold BlaP in order to generate amyloid-like fibrils from the 55Q chimera is reminiscent of that of proteolysis observed with some proteins associated with polyQ diseases. For example, nuclear inclusions from HD patients have been shown to contain Htt fragments including the expanded polyQ tract rather than the full-length protein [51]. It has been proposed that these fragments have a higher tendency to self-associate and thus may be crucial to initiate the aggregation phenomenon [28], [29]. Their greater propensity to aggregate is probably due to the removal by proteolysis of some of the steric hindrances otherwise exerted by the non-polyQ domains.

The protective effects mediated by the folded state of BlaP could also be due to the imposition of some conformational constraints on the polyQ tract. In a similar vein of what has been suggested by a number of experimental and computational studies [23], [52], [53], [54], we propose that the polyQ tract which extends from the solvent-exposed loop in BlaP exists as an ensemble of heterogeneous disordered conformations, in dynamic equilibrium with rare more structured conformational species, some of which are competent for amyloid fibril formation. The longer the polyQ repeat, the more dynamic it is and the wider the conformational space it can sample. As a consequence, more aggregation-prone conformations can be transiently sampled by longer polyQ tracts, thus explaining their higher propensity to aggregate. The length-dependent frequency at which the amyloid-competent conformations are visited, and thus the propensity of the polyQ tract to aggregate into amyloid-like fibrils, is further modulated by the conformational state of the β-lactamase moiety. Short polyQ stretches (≤30 residues) rarely or never access amyloid-competent conformations, even if the β-lactamase is in its unfolded state. In contrast, such conformations are equally sampled by the long polyQ tracts (≥79 residues) regardless of whether BlaP is native or unfolded. Finally, for polyQ stretches of intermediate lengths (i.e. 30–79 residues), the frequency at which amyloid-competent conformations are adopted critically depends on the structural integrity of BlaP. For instance, the presence of folded BlaP prevents the 55-glutamine tract from accessing conformational precursors to amyloid-like fibril formation. Conversely, the unfolding of the β-lactamase moiety would allow the 55-glutamine tract to interconvert more freely between various conformations and hence access, more frequently, conformations that are competent for amyloid-like fibril formation. The probability of accessing these conformations is however lower for the 55- than the 79-glutamine tract, and thus a lag phase is observed for BlaP(Gln)_55_ fibril formation. These results are consistent with previous reports showing the importance of the constraints imposed by adjacent sequences on the polyQ tract [23], [30]. For example, it was reported that the C-terminal addition of 10 or 11 prolines to polyQ peptides tends to decrease both aggregation rate and aggregate stability, and increases the threshold for fibril formation by disfavoring aggregation-competent conformations [23], [30]. The study of Darnell *et al.* strongly suggests that it is the formation of constraining polyproline type II helices by the C-terminal prolines that tips the balance in favor of aggregation-incompetent conformations [30]. Moreover, a recent study has shown that when a polyQ tract of pathological length is positioned between two SpA domains, it triggers slower aggregation than when it is located at the N-terminus of one SpA domain [34]. The authors proposed that the reduction of the aggregation rate is due to lower conformational freedom and higher steric constraints.

Results obtained for the other model proteins with an inserted polyQ tract can tentatively be analyzed in terms of the balance between the intrinsic propensity of polyQ tracts to trigger aggregation into amyloid-like fibrils and the constraints imposed by the host protein on the polyQ tract. It is interesting to note that the chimera of myoglobin containing 50Q aggregates into amyloid-like fibrils when incubated under conditions similar to those where BlaP(Gln)_55_ does not [18]. This observation suggests that steric/conformational constraints imposed on the polyQ tract in myoglobin are lower than those in BlaP. These lower constraints could, at least in part, originate from the fact that (i) the structure of myoglobin is perturbed by the insertion of the polyQ tract while the structure of BlaP is not and (ii) the loop in which the polyQ tract is inserted is significantly longer in myoglobin than in BlaP, essentially due to the addition of several amino acids from ataxin-3 at both sides of the polyQ tract [37]. In the case of apomyoglobin, the insertion of 38Q, the longest tract used in the aggregation studies, does not induce amyloid-like fibril formation. This observation suggests that a 38Q tract inserted into apomyoglobin is not long enough to access amyloid-competent conformations and/or to overcome the steric hindrances exerted by apomyoglobin.

Under native conditions, traces of dimeric species are observed for both BlaP and all chimeras with the highest amount being observed for wild-type BlaP (Table 1) which does not form amyloid-like fibrils. It is therefore very unlikely that the observed dimers act as seeds to facilitate fibril formation by BlaP(Gln)_79_ but not, for example, BlaP(Gln)_55_. The high molecular weight oligomeric species are however observed only for BlaP(Gln)_79_; since this protein is the only one to aggregate into amyloid-like fibrils under native conditions, the observed oligomers could indeed act as seeds of fibril formation and thus accelerate the process. A deeper characterization of the structural properties of the oligomeric species and of their role in the process of aggregation is under investigation. The potential of these species to act as seeds does not, however, invalidate the conclusions of the work. Indeed, this would purely imply that the constraints applied by the BlaP moiety act to prevent the formation of amyloid-like fibrils, at least in part, by preventing the formation of oligomeric species that are formed early on the pathway of aggregation.

### Conclusions

We have created and characterized a series of chimeras with 23, 30, 55 and 79 glutamines inserted into a solvent-exposed loop of a globular protein, the β-lactamase BlaP. The threshold number of glutamines above which the BlaP chimeras aggregate into amyloid-like fibrils critically depends on the structural integrity of the β-lactamase moiety. This result suggests that this threshold value results, at least in part, from a delicate balance between the intrinsic propensity of polyQ tracts to aggregate and the extrinsic protective conformational/steric constraints originating from BlaP. While it has been suggested that, in proteins associated with diseases, polyQ tracts are located in regions that are essentially unstructured and generally N- or C-terminal to a structured domain [35], several studies have shown that sequences flanking the polyQ tract could, however, adopt some elements of secondary structure [30], [55]. The latter could therefore exert constraints on the polyQ tract similar to those exerted by the BlaP moiety. The threshold for amyloid-like fibril formation by the BlaP chimeras under native conditions (>55Q) is higher than the highest threshold length observed for proteins associated with diseases (49 residues in atrophin-1, [5]). This observation suggests that the constraints applied to the polyQ tract by BlaP in its folded state are higher than those imposed by the proteins associated with polyQ diseases, probably because BlaP is a more structured and rigid scaffold. The use of this globular protein has however allowed us to produce and characterize, for the first time, a model protein with an inserted 79Q tract. Interestingly, our results clearly show that the structural integrity of BlaP, and thus the constraints imposed on such a long tract, has negligible impact on the propensity of the latter to mediate amyloid-like fibril formation. Based on these observations, we speculate that the modulating effects of the protein context on the aggregating properties of proteins associated with polyQ diseases could also be negligible when a particularly long polyQ tract is present. PolyQ tracts of 88–306 residues were reported for some proteins associated with polyQ diseases [1]; the aggregating properties of proteins with such long tracts could be, therefore, dictated essentially by the intrinsic propensity of the polyQ expansion to form intermolecular β-sheets.

Finally, this study demonstrates that BlaP is an appropriate scaffold to further investigate the delicate balance between the propensity of polyQ tracts to trigger aggregation and the modulating effects of the host proteins.

## Materials and Methods

### Molecular biology

A library of (CAG)_n_ double-strand DNA fragments was constructed by an overlapping PCR strategy [56], using the following oligonucleotides: 5′-(CAG)_13_-3′ and 5′-(CTG)_13_-3′. A PCR using Pfu DNA polymerase (Promega, Madison, WI, USA) was performed as follows: 3 min at 95°C, 30 sec at 94°C, 1 min at 55°C and 30 sec at 68°C; the last three steps were repeated 35 times. The library was purified from a 2% agarose gel and the extremities of each double-strand DNA fragment were blunt-ended with Pfu DNA polymerase and dNTPs at 68°C over a period of 30 min. The polyCAG DNA fragments were then dephosphorylated for 30 min at 37°C using calf intestinal alkaline phosphatase (Roche, Manheim, Germany). Finally, the polyCAG double-strand DNA library was inserted into the SmaI restriction site, which was previously introduced in the gene of BlaP carried by the constitutive expression vector pNYESBlaP [44]. In this vector, the gene of BlaP is followed by the nucleotide sequence coding for an additional C-terminal dipeptide glycine-proline and a (His)_5_ tag. The resulting library of expression vectors was used to transform *E. coli* DH5α cells (Invitrogen, Paisley, UK). Note that the β-lactamase activity which enables *E. coli* to become resistant to antibiotics can be used as a reporter to efficiently select clones producing soluble chimeras in which BlaP is correctly folded. Consequently, the transformed cells were plated on LB (Luria-Bertani) medium containing ampicillin (10 µg·mL^−1^, Sigma) in addition to spectinomycin (75 µg·mL^−1^, Sigma) for which the plasmid contains a resistance gene. The presence of an insert within the BlaP gene was checked by colony-PCR on more than 50 randomly selected transformants, and plasmids from about 10 colonies carrying an insertion were amplified and extracted. Their sequences were determined by the Sanger method at the GIGA GenoTranscriptomics technology platform (Liège, Belgium).

### Protein expression and purification

The proteins were expressed in *E. coli* JM109 strains (Promega). A 100 mL LB medium preculture, containing 75 µg·mL^−1^ of spectinomycin and 10 µg·mL^−1^ of ampicillin, was inoculated with the transformed cells and incubated at 37°C for approximately 7 hours. Two liters of modified TB (Terrific Broth) medium containing no glycerol, but supplemented with 75 µg·mL^−1^ of spectinomycin, 10 µg·mL^−1^ of ampicillin, 4.2 µM of biotin and 2.2 µM riboflavin, were inoculated with 5 mL of preculture and incubated overnight at 37°C. Periplasmic proteins were then extracted by osmotic shock as described by Vandevenne *et al.* [44]. The proteins of interest, which were expressed with a C-terminal (His)_5_ tag, were purified in a single step by metal chelate affinity chromatography, using a 20 mL Ni-PDC column (Affiland, Liège, Belgium). After loading the periplasmic extract, the column was washed successively with 60 mL of: (i) PBS (50 mM sodium phosphate, pH 7.5, containing 150 mM NaCl); (ii) PBS containing 2 M NaCl; (iii) PBS containing 10 mM imidazole. Proteins were eluted with a linear imidazole gradient (0–300 mM) in PBS. Enzymatically active fractions (probed with nitrocefin as the substrate) containing more than 95% of the protein of interest [as assessed by 15% (w/v) SDS-PAGE] were pooled. They were then either dialyzed four times against 15 L of milliQ water, lyophilized and stored at 4°C, or dialyzed two times against 15 L of PBS and stored at −20°C.

### Size-exclusion chromatography

A Superdex 200 GL 10/300 column (G.E. Healthcare, Uppsala, Sweden) was equilibrated with PBS buffer, pH 7.5. Solutions of BlaP and the four chimeras at 50 µM and 120 µM, and a solution of the chimera with 79 glutamines at 10 µM, in PBS, pH 7.5, were injected and eluted at a flow rate of 0.5 mL·min^−1^. Elution of protein was monitored by absorbance measurements at 280 nm. The column was calibrated using 7 protein standards: hen egg white lysozyme (Belovo, Bastogne, Belgium), chicken egg white albumin (Sigma, A2512), phosphorylase B from rabbit muscle (Sigma, P6635), β-galactosidase from *E. coli* (Sigma, 48275), aldolase from rabbit muscle (Sigma, A2714), catalase from bovine liver (Sigma, C40) and thyroglobulin from bovine thyroid (Sigma, T9145).

### N-terminal sequencing and mass spectrometry

N-terminal sequencing was performed using the Edman degradation procedure according to Han's protocol [57]. The molecular masses were determined using electrospray ionisation quadrupole time-of-flight mass spectrometry (ESI-QTOF-MS) at the GIGA proteomics platform (Liège, Belgium). The percentage of each species observed within the same sample injection run was estimated from the centroid value of peak.

### Quantification of the protein

The molar extinction coefficient (33000 M^−1^·cm^−1^ at 280 nm) of BlaP was determined experimentally using the BCA assay from Pierce (Rockford, IL, USA). This value was used for the determination of the concentrations of both wild-type and chimeric enzyme solutions.

### Enzymatic activity measurements

Kinetic parameters were determined at 30°C with cephalothin (Sigma C4520, 50–100 µM) as the substrate, in 50 mM sodium phosphate buffer, pH 7, using a UVIKON 860 spectrophotometer (Kontron Instrument, Zürich, Switzerland), as described by Matagne *et al.* [43].

### Fluorescence and circular dichroism measurements

Fluorescence data were acquired using either a Cary Eclipse spectrofluorimeter equipped with a Peltier-controlled holder (Varian, Mulgrave, Australia) or a LS50B spectrofluorimeter (Perkin-Elmer, Norwalk, CT, USA) and a 1 cm pathlength cell. CD measurements were performed on a Jasco J-810 spectropolarimeter (Jasco, Tokyo, Japan) equipped with a Peltier-controlled holder and using 1 mm and 1 cm pathlength cells for far-UV and near-UV measurements, respectively.

### Fluorescence and circular dichroism spectra of native proteins

All spectra were recorded at 25°C in PBS, pH 7.5, using protein concentrations of 4.6 µM for fluorescence and far-UV CD measurements, and 20 µM for near-UV CD measurements. Five fluorescence emission spectra were recorded in the 300–400 nm range (λ_exc_ = 295 nm, slit_exc_ = 2.5 nm, slit_em_ = 5 nm), at a rate of 600 nm·min^−1^ using the Cary Eclipse spectrofluorimeter, and averaged. Twenty CD spectra were acquired at a rate of 50 nm·min^−1^, both in the far-UV (200–250 nm) and near-UV (250–350 nm) regions, and averaged. The bandwidth and the response time were 1 nm and 0.5 sec, respectively. All protein spectra (fluorescence and CD) were corrected for the buffer contribution.

### Urea-induced unfolding experiments

Protein samples (4.6 µM) in PBS, pH 7.5, at various urea concentrations (VWR BDH Prolabo, 0 to 5.5 M by 0.1 M increments) were unfolded to equilibrium by incubation for *ca.* 16 h at 25°C. Unfolding was monitored by changes in intrinsic fluorescence (λ_exc_ = 295 nm, λ_em_ = 323 nm, slits_exc/em_ = 2.6 nm) using the LS50B spectrofluorimeter, and in far-UV CD signal at 222 nm (bandwidth = 1 nm, response = 4 sec), as described previously [58]. The background of the solutions (PBS buffer+denaturant) was subtracted from the fluorescence and CD signals. Urea concentrations were determined from the refractive index measurements [59] using a R5000 refractometer from Atago (Tokyo, Japan). Moreover, other protein samples were denatured for 3 hours at 25°C in 5.5 M urea (under these conditions, all the investigated proteins have been shown to be completely unfolded) and renatured (for *ca.* 16 h) by dilution to different urea concentrations (from 5.5 to 0.55 M). The reversibility of the unfolding transitions was demonstrated by comparing the fluorescence and CD signals recorded with these samples to those obtained for the samples unfolded at similar urea concentrations. Equilibrium unfolding curves were analyzed on the basis of a two-state model (N 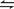 U), as previously described [58], [60], [61], [62].

### Heat-induced unfolding

Heat-induced unfolding was monitored by the changes in the intrinsic fluorescence intensity (λ_exc_ = 295 nm, λ_em_ = 323 nm, slits_exc/em_ = 5 nm) using the Cary Eclipse spectrofluorimeter, and in far-UV CD signal at 222 nm (bandwidth = 1 nm, response = 4 sec). The protein concentration was 4.6 µM in PBS, pH 7.5, and mineral oil was added on top of the samples to limit solvent evaporation. The temperature was increased from 25 to 85–90°C at a rate of 0.5°C·min^−1^; the fluorescence and the CD data were acquired every 0.5°C. The temperature in the cell was measured with a PT200 thermocouple (IMPO Electronic, Olgod, Denmark). The reversibility of the heat-induced unfolding was assessed by monitoring the changes in the fluorescence and CD signals upon cooling the sample down to 25°C at a rate of 0.5°C·min^−1^. Data were analyzed on the basis of a two-state model (N 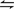 U), as described by El Hajjaji *et al.* [63].

### Aggregation kinetics

A series of tubes containing 100 µL of 110 µM protein in PBS, pH 7.5, containing 0.2% sodium azide, were incubated either at 37°C in the absence of urea or at 25°C in the presence of 1.85 or 3.5 M urea. Airtight tubes (Multiply Safecup, Sarstedt, Nümbrecht, Germany) were used to limit evaporation. At selected times, one tube was removed and centrifuged for 50 min at 12000 rpm. The supernatant was used to determine the quantity of soluble protein by absorbance measurements at 280 nm and the protein integrity was demonstrated by SDS-PAGE analysis. Unless otherwise stated, the aggregation time-courses were repeated three times with proteins originating from different production and purification batches. For one of the aggregation time-courses of each protein, aliquots of samples at the initial (T_0_) and end (T_f_) time-points were taken (in triplicate) before centrifugation for thioflavin T (ThT) fluorescence measurements; they were kept frozen until analysis. Samples at T_f_ were also analyzed by transmission electron microscopy.

### Thioflavin T (ThT) fluorescence measurements

To 5 µL protein sample was added 1.5 mL of 10 mM sodium phosphate buffer, 150 mM NaCl, 50 µM ThT (Sigma, T3516), pH 7. Ten fluorescence emission spectra were recorded (using the Cary Eclipse spectrofluorimeter) at 25°C with stirring in the 450–600 nm range (λ_exc_ = 440 nm, slit_exc/em_ = 5 nm) at a rate of 1200 nm·min^−1^, averaged, and corrected for the background fluorescence of the ThT solution alone.

### Transmission electron microscopy

Samples were left for 4 min on carbon-coated 400-mesh copper grids, before being stained for 1 min with 2% uranyl acetate (w/v). The grids were then washed once with 2% uranyl acetate and finally, three times with milliQ water. Images were recorded on a Philips CEM100 transmission electron microscope operating at 100 kV.

### X-ray fibre diffraction

Fibril suspensions were centrifuged and the pellet subjected to two washing cycles in milliQ water; between each cycle, the suspension was centrifuged and the supernatant discarded in order to remove any trace of soluble protein, buffer, or urea. The fibrils were then aligned using a modification of the stretchframe method as previously described [64]. X-ray diffraction data were collected at room temperature for *ca.* 10 min on a Bruker AXS FR591 diffractometer with Cu Kα radiation with a wavelength of 1.5418 Å and equipped with a MARDTB 345 mm image plate detector. The sample-to-detector distance was 300 mm.
